# Antimicrobial proteins and peptides in pregnancy: Guardians of the maternal–fetal frontier

**DOI:** 10.3389/fimmu.2026.1783841

**Published:** 2026-05-13

**Authors:** Andrea Olmos-Ortiz, Bruno Rivas-Santiago, Oscar González-Muñíz, Daniel Ortuño-Sahagún, Ismael Mancilla-Herrera, Cecilia Helguera-Repetto, Verónica Zaga-Clavellina

**Affiliations:** 1 Departamento de Inmunobioquímica, Instituto Nacional de Perinatología, Ciudad de México, Mexico; 2 Unidad de Investigación Biomédica de Zacatecas-IMSS, Instituto Mexicano del Seguro Social Zacatecas, Zacatecas, Mexico; 3 Laboratorio de Neuroinmunobiología Molecular, Instituto de Neurociencias Traslacionales, Universidad de Guadalajara, Guadalajara, Mexico; 4 Subdirección de Investigación Biomédica, Instituto Nacional de Perinatología, Ciudad de México, Mexico; 5 Dirección de Investigación, Instituto Nacional de Perinatología, Ciudad de México, Mexico

**Keywords:** alpha defensins, amniotic fluid, antimicrobial proteins and peptides, beta defensins, cathelicidin, fetal membranes, immune privilege, placenta

## Abstract

The success of human pregnancy relies on the precise synchronization and adaptation of the maternal immune system, which, together with fetal tissues, establishes a tolerogenic intrauterine environment while maintaining the capacity to mount effective immune responses. At the maternal–fetal interface, multiple physical and immune mechanisms coordinate the recognition and control of pathogens that could jeopardize pregnancy. Ascending infections from the lower genital tract can reach the uterine cavity, infect the fetal membranes (chorioamnionitis), and invade the amniotic fluid, triggering a proinflammatory response strongly associated with adverse outcomes. Both maternal and fetal compartments deploy several defense strategies, among which antimicrobial proteins and peptides (AMPs) play a central role. These small, pleiotropic molecules, produced mainly by epithelial surfaces and inflammatory cells, exhibit broad-spectrum antimicrobial and immunomodulatory activities, constituting a key component of the first line of defense at the maternal–fetal interface. This review summarizes the principal AMPs produced by the placenta, fetal membranes, decidua, maternal reproductive tract, and fetal tissues, describes their mechanisms of action, factors regulating their expression, and explores their role in both physiological and pathological processes.

## 1 Introduction

Pregnancy depends on a dynamic and highly specialized communication network among three key participants: the mother, the fetus, and the extraembryonic tissues, including the placenta and fetal membranes. This triad continuously adapts to metabolic, anatomical, endocrine, and immune changes throughout the three trimesters of gestation, to prevent rejection and establish an immunologically privileged site. Effective communication within this system is essential for responding to internal and external stimuli that may threaten fetal development. Additional components of the maternal-fetal interface (MFI), including the decidua, maternal leukocytes, and amniotic fluid, also play active roles in this protective network, contributing to fetal defense and, the overall success of pregnancy.

A major threat during pregnancy is intrauterine bacterial infection, which can lead to premature rupture of membranes (PROM), preterm birth (PTB), and neonatal complications associated with prematurity, such as fetal growth restriction, birth asphyxia, admission to the neonatal intensive care unit, fetal inflammatory response syndrome and, in the most severe cases, stillbirth or neonatal death (1). To counteract infections, tissues at the MFI have evolved robust physical and immunological mechanisms of microbial defense (2). The first line of protection consists of physical barriers that prevent ascending pathogens from reaching the upper reproductive tract. Beyond these structural defenses, immunological barriers at the MFI integrate multiple strategies to safeguard the fetus, including the production of antimicrobial proteins and peptides (AMPs).

**Figure 1:**
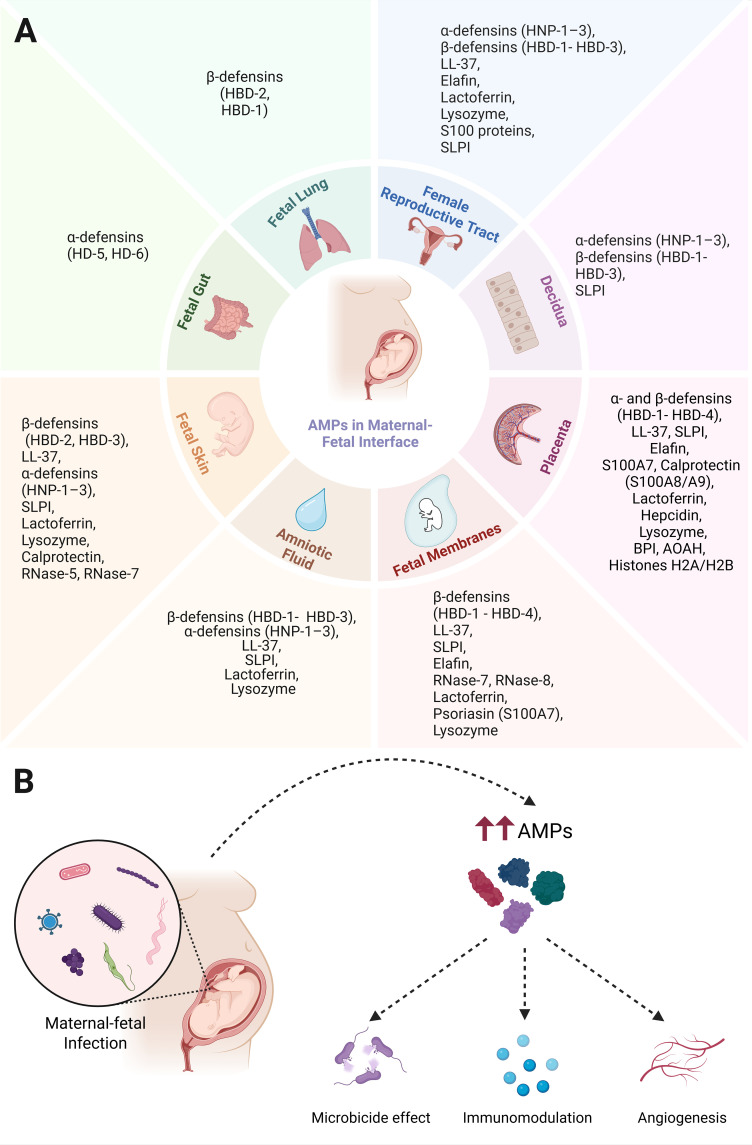
Antimicrobial defense at the maternal–fetal interface. **(A)** all tissues of the maternal–fetal interface (MFI) actively produce antimicrobial peptides and proteins (AMPs), each displaying compartmentalized yet overlapping protective functions. **(B)** AMPs play a central role in defending and protecting the MFI against a wide range of microorganisms that can jeopardize both fetal and maternal health. Beyond this critical function, AMPs also regulate immune responses, angiogenesis, and oxidative stress, among other processes, highlighting their versatility in both physiological and pathological pregnancies. Created in https://BioRender.com.

This review provides an integrated and comprehensive overview of AMPs as fundamental components of innate immune defense at the maternal–fetal interface. We summarize current knowledge on the structural and immunological barriers protecting the intrauterine environment, describe the diversity and function of major AMPs families, such as defensins and cathelicidins, and examine their compartmentalized expression in the decidua, maternal immune cells, placenta, fetal membranes, amniotic fluid, and fetal tissues. Additionally, we discuss their immunomodulatory roles, their regulation under physiological and infectious conditions, and highlight emerging perspectives for future research (**Figure 1**).

### 1.1 Protective mechanisms at the maternal-fetal interface: physical barriers

The MFI is protected by a coordinated network of physical and immune mechanisms that prevent microbial invasion while preserving fetal tolerance. In the lower reproductive tract, the vaginal environment serves as the first line of defense through its acidic pH, maintained primarily by lactic acid-producing *Lactobacillus* species (3), as well as by the integrity of the epithelial tight junction barrier and the production of cervicovaginal mucus, which trap and facilitate the clearance of microorganisms (4, 5). During pregnancy, the cervical mucus plug further reinforces this barrier by preventing ascending infections into the uterine cavity and maintaining the immune privilege (6).

Beyond the vagina and cervix, the placenta and fetal membranes provide a robust physical barrier that separates the fetus from maternal circulation and potential pathogens. The syncytiotrophoblast, a continuous multinucleated layer lacking intercellular junctions, is a key structural defense against microbial entry. This barrier spans approximately 5 m^2^ at mid-gestation and expands to 11–12 m^2^ at term (7). In addition, extracellular matrix components –including various collagen types, laminin, microfibrils, elastin, fibronectin, actin and integrins–, are present in the decidua, placenta and fetal membranes and contribute to tissue integrity and mechanical resistance, thereby limiting pathogen dissemination (8). Notably, loss of fetal membrane integrity, structural integrity, and mechanical resistance increases the risk of PROM, which may allow pathogens to enter the amniotic cavity, increasing both maternal and fetal morbidity and mortality. Consequently, when mechanical stability is compromised, innate immune responses become crucial for preventing microbial ascent and maintaining fetal well-being.

### 1.2 Innate immunity taking control of the MFI

Beyond their role as physical barriers, tissues at the MFI display coordinated innate immune activity that not only protects the fetus but also initiates integrated mechanisms that contribute to the establishment of a tolerogenic environment. Among these tissues, the decidua, fetal membranes, and placenta play central roles in immunological surveillance. The placenta, in particular, has been proposed as a pivotal component of the innate immune system, responding to diverse challenges such as infection, hypoxia, and nutritional imbalances (9–11).

To fulfill this role, human trophoblasts express various pattern recognition receptors (PRRs), including toll-like receptors (TLRs), enabling the sensing of pathogens and the secretion of inflammatory mediators such as chemokines, and cytokines, while simultaneously restricting microbial transmission. Notably, all 10 TLR types have been identified in trophoblasts throughout gestation, following a temporally regulated pattern (12). Additionally, local placental synthesis of complement proteins (13) and the recruitment of immune cells, including natural killer (NK) cells, macrophages, and dendritic cells (DC) to decidua (14–16), supports pathogen clearance while maintaining immune tolerance.

Innate immune cell populations at the MFI must be tightly regulated during pregnancy. Although innate immunity predominates in this environment, its crosstalk with adaptive responses is essential to maintain immune tolerance. Increased infiltration of CD4^+^ and CD8^+^ T cells in spiral arteries has been reported in hypertensive disorders of pregnancy, together with alterations in the local cytokine milieu (17). This suggests that dysregulated adaptive immune activation may disrupt the finely tuned balance required at the interface, representing a clinically relevant scenario in which innate and adaptive immune responses converge and potentially contribute to adverse outcomes.

The innate immune response at the MFI is supported not only by immune cells, cytokines and chemokines, but also by AMPs. These molecules provide a first-line defense by exerting direct microbicidal activity and modulating host immunity (18). Thus, they represent a critical link between barrier function and innate immunity at the MFI.

## 2 Antimicrobial peptides: always there

AMPs are small amphipathic peptides that provide a rapid innate immune response to microbial invasions, disrupt microbial growth, and offer essential protection against bacterial, viral, and fungal pathogens. They exhibit broad-spectrum activity, and complete bacterial resistance to AMPs is rare (19). In addition to disrupting bacterial membranes, AMPs participate in key physiological processes such as fertilization, implantation, and fetal development, highlighting their broader relevance during pregnancy.

Based on size, antimicrobial peptides can be classified as ultra-short (2–10 amino acids: aa), short (10–24 aa), medium (25–50 aa), or long (50–100 aa), with molecular weights usually ranging from 1.5 to 6 kDa. At physiological pH, they generally have a net positive charge (+2 to +9) due to their high content of basic amino acids, such as arginine, lysine, and to a lesser extent, histidine (20). More than twenty biological functions have been identified for AMPs, including antibacterial, antiviral, antifungal, antiparasitic, antibiofilm, antitoxin, antiendotoxin, antidiabetic, anti-inflammatory, antioxidant, wound healing, chemotactic, spermicidal, insecticidal, ion channel inhibition, and protease inhibition, among others.

As of January 2025, the Antimicrobial Peptide Database (APD3) listed 5,099 peptides: 3,306 natural AMPs with experimentally validated activity from the six kingdoms of life [bacteria (410), archaea (5), protists (8), fungi (29), plants (268), and animals (2,580)], 1,299 synthetic AMPs, and 231 computationally predicted AMPs (21). Recently, the AMPphere database cataloged more than 860,000 non-redundant peptide sequences in prokaryotes derived from metagenomic data and high-quality microbial genomes; some of these have been tested and shown effective antimicrobial activity against pathogenic bacteria (22). Therefore, AMPs are ubiquitously present in all domains of life and have critical functions beyond microbial lysis. For this review, we focus on natural AMPs produced by tissues of the MFI during human pregnancy.

Natural AMPs from mammals can originate by two main routes: proteolysis (23) or direct transcription and translation from the genome (24). Prokaryotes and fungal eukaryotes can also produce active AMPs through non-ribosomal synthesis with non-proteinogenic amino acids (25).

**Figure 2:**
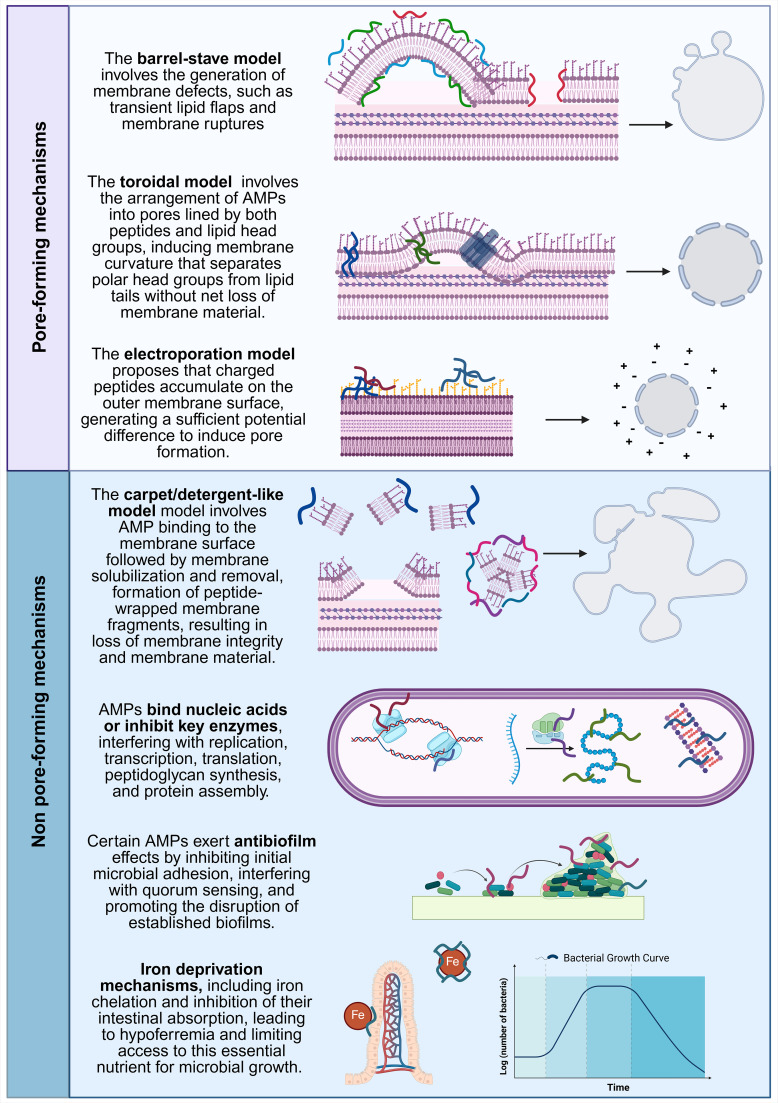
Multifaceted mechanisms of AMPs. AMPs act through two main, partially overlapping mechanisms (1): membrane-disruptive (Pore-forming) and (2) non-membrane disruptive (non-pore forming) pathways. Pore-forming mechanisms include the barrel-stave model, where peptides insert and oligomerize into transmembrane channels; the toroidal model, in which pores are lined by both peptides and lipid head groups; and electroporation-like effects driven by peptide-induced membrane potential. Non-pore forming mechanisms, include the carpet (detergent-like) model, leading to membrane disintegration, as well as intracellular targeting (e.g. nucleic acids and key enzymes), antibiofilm activity, and iron sequestration. These mechanisms are not mutually exclusive and often act cooperatively to enhance antimicrobial efficacy. Created in https://BioRender.com. Models adapted from (27, 106, 173).

AMPs interact with pathogens through multiple sophisticated mechanisms, providing robust defense against bacteria, viruses, fungi, and parasites. Their activity relies primarily on electrostatic interactions: most AMPs are cationic (positively charged) and amphipathic, enabling them to bind to negatively charged components of microbial membranes, such as phospholipids, including phosphatidylserine, cardiolipin and phosphatidylglycerol; bacterial membranes also contain unique lipid components: lipopolysaccharides (LPS) in Gram-negative bacteria or teichoic acids in Gram-positive bacteria (26). This lipid and antimicrobial interaction destabilizes membranes, promotes pore formation, and ultimately leads to cell lysis. Several models have been proposed to describe this process, including aggregate, toroidal pore, barrel-stave, and carpet or detergent-like mechanisms (27–30) (**Figure 2**). Some AMPs can also synergize with mammalian histones to enhance large membrane pore formation (31).

Beyond membrane disruption, AMPs can penetrate microbial cells and target intracellular processes. They may bind nucleic acids or inhibit key enzymes, interfering with replication, transcription, translation, peptidoglycan synthesis, and protein assembly (32–34). These actions impair essential cellular functions and compromise pathogen survival.

AMPs also have important immunomodulatory functions. They recruit and activate immune cells, stimulate cytokine production, and promote wound healing and resolution of inflammation (32). Furthermore, AMPs inhibit biofilm formation, a major microbial survival strategy that protects bacteria from host defenses and antibiotics. By disrupting biofilms, AMPs make pathogens more susceptible to immune clearance and antimicrobial therapies (26).

AMPs often act synergistically with other host defense molecules, such as lysozyme and antibodies, enhancing overall antimicrobial efficacy (32). These diverse mechanisms highlight the versatility and efficiency of AMPs as key components of innate immunity.

A distinguishing feature of AMPs is their broad diversity in sequence, conformation, and three-dimensional structure. Based on these characteristics, they are classified into four groups: α-helical linear peptides, peptides with β-sheet conformation and disulfide bridges, cyclic peptides, and peptides with long flexible loop structures. Approximately 16.6% of all AMPs have an α-helical conformation. The cathelicidin family is a significant member of this category, and most are α-helical (23–37 amino acids). Peptides with a β-sheet core and three disulfide bridges between six conserved cysteine residues are referred to as defensins (classified into α-, β-, and θ-defensins) and, along with cathelicidins, comprise one of the most studied peptide families in vertebrates.

### 2.1 The cathelicidin family

Over the past two decades, the cathelicidin family —particularly the human cathelicidin— has attracted considerable attention for its multifunctional roles in host defense, immune modulation, and tissue homeostasis. Members of this family include β-hairpin peptides, protegrins, indolicidin, and proline-rich porcine peptides; however, humans express only cathelicidin. This AMP is encoded by the CAMP gene, which produces cathelicidin hCAP-18, later cleaved into the cationic active product known as LL-37 (35).

LL-37 is named for its primary structure, which begins with two leucine (L) residues and consists of 37 amino acids: LLGDFFRKSKEKIGKEFKRIVQRIKDFLRNLVPRTES. At neutral pH, 16 out of the 37 amino acids are charged. Of these, 6 lysines (K) and 5 arginines (R) contribute positive charges, while 3 glutamic acids (E) and 2 aspartic acids (D), contribute negative charges, resulting in a net charge of +6. This cationic nature, along with its amphipathic alpha-helical structure, underlies its interaction with microbial membranes. The amphipathicity results from the spatial segregation of hydrophobic and hydrophilic amino acids along the helix, allowing LL-37 to interface efficiently with lipid bilayers. These structural features are central to the proposed mechanism of action of LL-37. The peptide is attracted to the negatively charged components of bacterial membranes, such as lipoteichoic acids in gram-positive bacteria or lipopolysaccharides in gram-negative bacteria. After the initial electrostatic interaction, LL-37 inserts into the lipid bilayer, leading to peptide aggregation and membrane disruption. This process has been modeled by the “barrel-stave” mechanism, in which multiple peptides align perpendicularly to the membrane, forming transmembrane pores that compromise membrane integrity and result in microbial lysis (33, 36). Reduced expression of cathelicidin is associated with increased susceptibility to infections, particularly at mucosal surfaces, where the innate immune system relies heavily on antimicrobial peptides for first-line defense.

LL-37 expression increases in response to various pathogens and has been detected during infections with *Staphylococcus aureus* (37), *Mycobacterium tuberculosis* (35), *Candida albicans* (38), *Escherichia coli* (39), and Group A *Streptococcus* (40). LL-37 also protects against *Staphylococcus aureus* and *Candida albicans* (26, 32). Beyond its direct antimicrobial action, LL-37 aids infection resolution by modulating inflammatory signaling, promoting neutrophil recruitment, stimulating neutrophil extracellular tramps (NETs) formation, enhancing phagocytosis, supporting DC differentiation, and strengthening epithelial barrier integrity (41–43).

### 2.2 α- and β-defensins

Classification into α-, β-, and θ-defensins is based on amino acid homology and cysteine residue connectivity. Defensins contain both α-helical and β-sheet structures. Humans express only α- and β-defensins; α-defensins are further subdivided into enteric and myeloid defensins. NK cells and primarily neutrophils express four types of α-defensins, known as human neutrophil peptides (HNP1-4), which are stored in granules. These granules can fuse with phagosomes and release the peptides onto the surface of pathogens (44). Paneth cells are the main producers of enteric α-defensins (HD-5 and -6). HD-5 is effective at killing gram-negative bacilli (e.g., *Escherichia coli, Staphylococcus aureus*, and *Enterobacter aerogenes*), while HD-6 has poor direct antimicrobial effects but can immobilize bacteria by self-assembling into a nanonet (45, 46). In contact with intestinal mucus, both HD-5 and HD-6 can generate active fragments that act against pathogens and modulate gut microbiota composition (46).

β-defensins are expressed in all vertebrates and are synthesized mainly by epithelial cells. In humans, 15 beta defensin genes (hBD) are distributed across four chromosomes, with the most studied defensins (hBD-1, -2, -3, -4, -5, -6, -7, and -9) located on chromosome 8. Unlike α-defensins, β-defensins can be released directly from the cytoplasm to the extracellular compartment by either stimulated or constitutive expression. These peptides can form dimers or multimers, increasing their ability to bind pathogen membranes and enhance bacterial lysis (47). HBD-2, in particular, shows broad activity against *E. coli* and group B *Streptococcus* and can also bind viral glycoproteins to block infection (18, 26).

θ-defensins have been found only in circulating neutrophils, monocytes, and immature and mature myeloid cells in the bone marrow of rhesus macaques (48) and olive baboons (49). Their structure differs from that of α- and β-defensins, as they are macrocyclic peptides rich in cysteine motifs, making them more resistant to high salt concentration and protease activity (50). They play a central role in the microbicidal activity of neutrophil granules (48).

## 3 The MFI as a coordinated antimicrobial defense zone

AMPs are produced by multiple cell types at the MFI, including villous and extravillous trophoblasts, decidual cells, amniotic epithelial cells, trophoblasts from the choriodecidua, maternal leukocytes and lymphocytes, as well as by fetal tissues such as the lungs, gut, and skin. Within this complex microenvironment, AMPs perform essential functions to protect the fetus from bacterial, viral, and fungal infections. Although present in small quantities (usually in the pg-ng range), they can act cooperatively and synergistically, optimizing their effects both locally and peripherally (51, 52).

**Figure 3:**
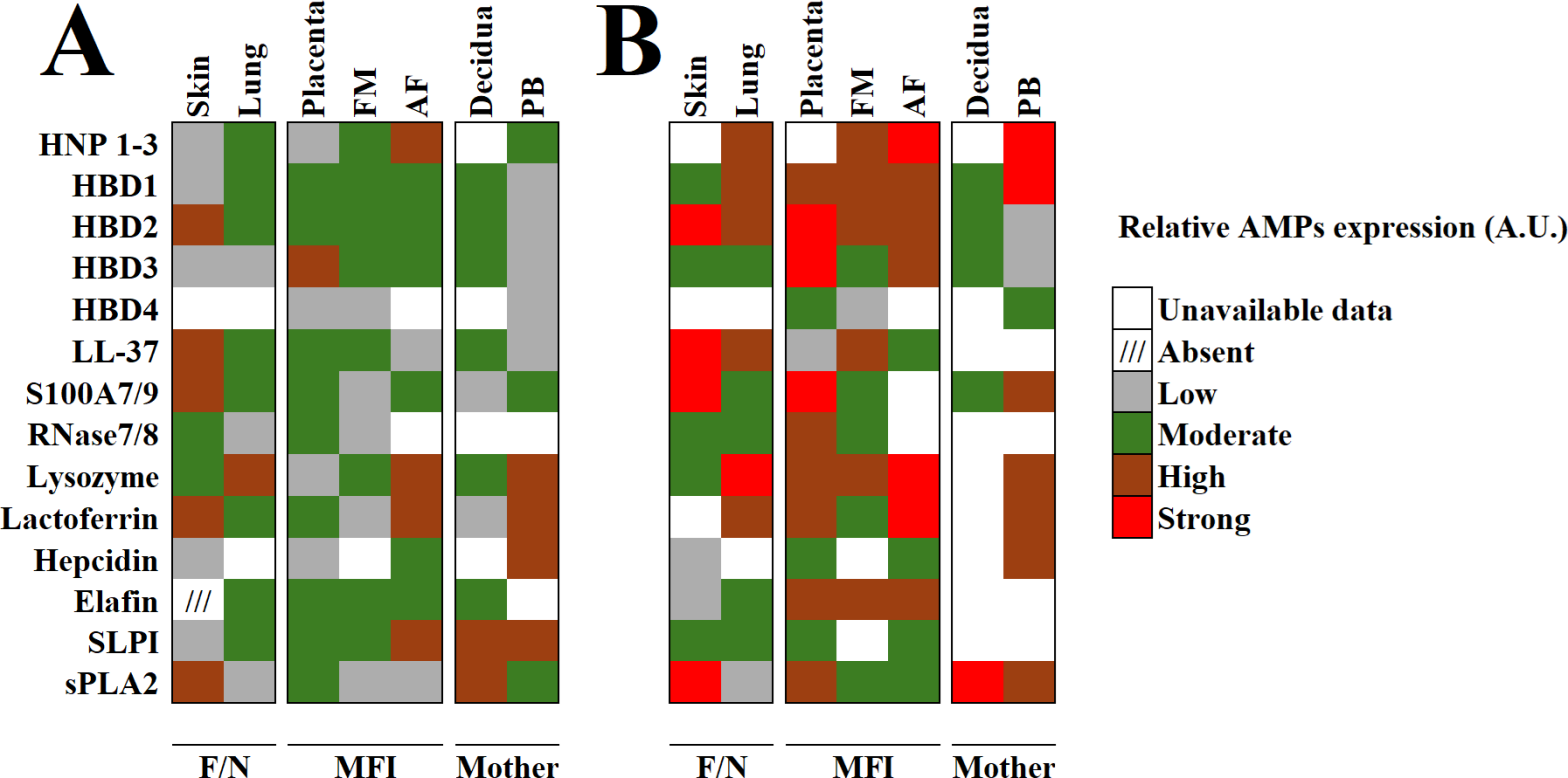
Heat map showing the basal and induced expression of AMPs by maternal, fetal, and maternal-fetal interface components. **(A)** basal expression of major antimicrobials. **(B)** expression of major antimicrobials during infection (chorioamnionitis, intra-amniotic infection, or infection-related PROM) or inflammation (LPS, TNF-α, IL-1α, IL-1β). data on gene expressions, protein content, protein secretion, or serum/fluid levels from human reports were integrated and expressed in arbitrary units for comparative purposes. AF, amniotic fluid; FM, fetal membranes; F/N, fetus or neonate; MFI, maternal-fetal interface; PB, peripheral blood at term. Skin data includes reports from vernix caseosa and primary human keratinocyte cells. Lung data includes reports from tracheal aspirates, bronchoalveolar lavage fluids, and primary tracheobronchial cell cultures. References for: Skin (55, 134, 157, 158, 174–181), Lung (161, 182–191), Placenta (66, 79, 92, 94, 98, 111, 119, 124, 192–198), Fetal membranes (51, 52, 58, 128, 130, 138, 152, 167, 198–203), Amniotic fluid (131, 146–148, 156, 191, 203–209), Decidua (80, 86, 196, 202, 210–215), Maternal peripheral blood (191, 194, 203, 205, 216–224).

Dysregulated AMPs expression in the MFI has been linked to adverse outcomes, including preterm birth, preeclampsia, and impaired fetal development (53–57). Understanding the temporal and spatial dynamics of AMPs expression at the MFI may inform therapeutic strategies to prevent or treat pregnancy-related infections, strengthen placental defense mechanisms, and improve maternal and fetal health (58–61). In this review, we present an integrative approach to the expression and levels of AMPs in the tissues and fluids of the MFI, the mother, and the fetus under both basal and infectious or inflammatory conditions (**Figure 3**). All components provide overlapping protective strategies to ensure fetal protection and minimize maternal morbidity.

While their primary roles are antimicrobial activity and immunomodulation, AMPs also contribute to broader physiological processes at the interface. For example, human AMPs are associated with fetal development and immune system maturation (62). They also play protective roles during neurodevelopment; HBD-2, for instance, is induced in fetal brain cells by bacterial components, highlighting its role in protecting neuronal development against bacterial pathogenesis (63). In addition, AMPs support the maternal gut barrier during pregnancy and facilitate nutrient absorption. Reduced lysozyme gene expression in Paneth cells, for example, may impair maternal nutrition, and negatively affect fetal growth (64).

AMPs synthesis can be compromised by maternal conditions such as gestational diabetes and obesity, and may also be affected by micronutrient deficiencies during pregnancy —particularly vitamin D, and to a lesser extent vitamins A and E, as well as zinc— (65–72). Potential strategies to address AMPs deficiency during pregnancy include nutritional interventions, vitamin D supplementation, probiotics, and lifestyle modifications that enhance immune function. Moreover, synthetic AMPs therapies —though still experimental—, show promise for preventing pregnancy-related infections and complications (73, 74). Understanding where these peptides exert their protective functions during pregnancy is essential. One of the key sites of AMPs activity is the female reproductive tract and the decidua.

### 3.1 Female reproductive tract and decidua. A surveillance system always alert

Like the respiratory system and gastrointestinal mucosa, the female reproductive tract (FRT) is susceptible to invasive and opportunistic pathogens. Microbial invasion of the FRT can lead to fertilization failure, miscarriage, ectopic pregnancy, preterm delivery, and congenital or neonatal infections (75). The distribution of antimicrobials in the FRT is extensive and includes HNP-1-3, β-defensins, LL-37, elafin, lactoferrin, lysozyme, S100 proteins, secretory leukocyte protease inhibitor (SLPI), and C-type lectins, which perform several functions throughout a woman´s life, including during pregnancy.

α- and β-defensins are the most highly expressed AMPs in the FRT, according to transcriptomic data in the GEO database (76). Under basal conditions, they are present in the cervical and vaginal epithelium, but their expression can be markedly upregulated in response to infection (77, 78). The α-defensins HNP 1-3, produced by leukocytes (including neutrophils, eosinophils, NK cells, monocytes, and DC), are predominantly expressed in the lower FRT (vagina and ectocervix), and form a critical defense axis during pregnancy. In contrast, β-defensins (HBD-1, -2, and -3) are preferentially expressed along both the upper (endocervix, uterus, and fallopian tubes) and lower FRT (76).

AMPs expression in the FRT is influenced by several factors, including hormonal levels, age, vaginal microbiota, and localized or systemic inflammation (79, 80). During the proliferative (follicular) phase, the estrogen peak enhances endometrial HBD-4 and HD5 gene expression, as well as, to a lesser extent, calprotectin and lactoferrin (81). In contrast, the onset of the secretory (luteal) phase, characterized by rising progesterone levels, is associated with increased expression of cervicovaginal HBD-2 and HBD-3, as well as endometrial SLPI, HD5, HBD-1, and HBD-3 (76, 81–84). In the late secretory phase, HD5 and HBD-3 reach their highest expression levels in the endometrium. This cyclic up-regulation of peptides has been linked to enhanced protection during the “window of vulnerability” associated with menstrual bleeding (84).

As pregnancy begins, this hormonally driven antimicrobial environment becomes essential for establishing a receptive and infection-resistant endometrium. In early pregnancy, a critical event is the implantation of the blastocyst into the maternal endometrium. This process requires an active immune response, precise recruitment of leukocytes to the endometrium, and hormonal signals that transform stromal endometrial cells into the nutritive niche of the decidua (85). First-trimester decidual cells secrete SLPI, whereas midluteal endometrial stromal cells produce HNP1–3 and HBD-1; these peptides may therefore contribute to generating an antimicrobial environment that protects the blastocyst and favors implantation (86, 87). However, other defensins do not appear to participate in implantation or early placentation (87). Instead, decidualization is essential to sustain HBD-1, HBD-2, and HBD-3 secretion in response to bacterial infection, whereas non-decidualized endometrial stromal cells show reduced HBDs production under the same conditions (80).

### 3.2 Maternal immune cell–derived AMPs

Immune cells play a central role in regulating AMPs expression throughout the body, including the MFI during pregnancy. Among these cells, neutrophils are the primary producers of alpha defensins and cathelicidin, releasing these peptides to directly eliminate pathogens during infection. Neutrophils also secrete cytokines and chemokines that recruit and activate other immune cells, thereby modulating AMPs production in the local environment both directly and indirectly (88).

Macrophages contribute by producing proinflammatory cytokines such as IL-1β and TNF-α, which upregulate AMPs expression in epithelial and other tissue-specific cells. Through phagocytosis, macrophages help control microbial load, influencing the local demand for AMPs (89).

NK cells regulate AMPs expression by releasing IFN-γ, a key factor in antibacterial immune defenses. By targeting and lysing infected cells, NK cells further contribute to pathogen clearance (90). DC serves as key mediators between the innate and adaptive immune systems. They influence AMPs levels by producing cytokines that modulate the immune response and by presenting antigens to T cells. Among T cell subsets, Treg cells help maintain immune homeostasis by producing anti-inflammatory cytokines such as IL-10, which can suppress excessive inflammation and prevent AMPs overexpression. Conversely, Th1 and Th17 cells secrete IFN-γ and IL-17, respectively, promoting AMPs production in response to microbial challenges (91).

These interactions highlight the dynamic and finely tuned regulation of AMPs by the immune system and their complex crosstalk with other tissues at the MFI, ensuring robust antimicrobial defense while preserving immunological balance.

### 3.3 Placenta

The placenta is not only a physical selective barrier but also an active immunological organ that produces and secretes a wide array of AMPs, including defensins, LL-37, SLPI, elafin, S100A7, calprotectin, lactoferrin, hepcidin, lysozyme, bactericidal/permeability-increasing protein (BPI), acyloxyacyl hydrolase (AOAH), and histones H2A and H2B. Their expression is temporally and spatially regulated, reinforcing the placental barrier and preventing pathogen transmission from maternal blood to the fetus (79, 92–94). Beyond their antimicrobial activity, these molecules also regulate other processes, including angiogenesis. For example, *in vitro* stimulation of endothelial cells with HBD-2 promotes proliferation and the formation of capillary-like structures independently of vascular endothelial growth factor (VEGF) signaling (95). In contrast, BPI (in addition to its neutralizing effect on lipopolysaccharide) and HNP1–3 demonstrate antiangiogenic and apoptotic effects in endothelial cells (96, 97). These findings suggest that placental antimicrobials, at least α-defensins, HBD-2, and BPI, may locally regulate capillary villi growth and arborization.

In addition to estrogens and progestins (76), other steroids are key regulators of AMPs expression in the placenta. Calcitriol stimulates the expression of HBD2, HBD3, and LL-37 in primary villous trophoblast cultures (98). Experimental stimulus with testosterone exerts a sexually dimorphic effect by reducing calcitriol-dependent cathelicidin expression in primary trophoblasts, leading to lower LL-37 levels observed in placentas and umbilical cord serum from male neonates (99). This difference may contribute to the higher susceptibility of male neonates to infections, an effect further exacerbated in pregnancies complicated by urinary tract infections (100). Importantly, during late pregnancy, LL-37 is transferred from mother to fetus via the placenta in a neutrophil-dependent manner, thereby enhancing immune defenses at birth and during the neonatal period (101). This finding is consistent with the progressive increase of cord blood LL-37 levels across gestation, whereas their decline correlates with recurrent infections and preterm complications, such as bronchopulmonary dysplasia (102). Moreover, placental LL-37 expression has been positively associated with neonatal outcomes; its gene expression correlates with birth weight and, in pregnancies complicated by urinary tract infections, with gestational age, suggesting a protective role against preterm birth (100).

Among the less studied placental AMPs are the antileukoproteases SLPI and elafin, whose primary function is to neutralize leukocyte-derived proteases, such as the neutrophil elastase, cathepsin G and proteinase 3, thereby preventing tissue damage caused by excessive protease activity (103). In addition, both peptides exhibit antibacterial and anti-inflammatory properties (104, 105) and are experimentally upregulated by inflammatory stimuli such as IL-1, TNF-α, or LPS in primary placental trophoblast cells (79, 106). Lysozyme, another placental AMPs, contributes to host defense by hydrolyzing bacterial cell walls (106, 107).

Other amphipathic peptides produced by the placenta include hepcidin and lactoferrin (108–113), the latter also being a major component of human milk. Both exert antimicrobial effects through direct electrostatic interactions with microbial membranes, as described previously, and by indirect iron deprivation. Specifically, hepcidin reduces intestinal iron absorption, while lactoferrin chelates iron, inducing hypoferremia and depriving microbes of this essential element for growth (106, 114). Notably, lactoferrin from human milk has been shown to inhibit the growth of *Listeria monocytogenes in vitro* (115, 116), suggesting a possible placental protective role for their hematogenous infection.

Calprotectin, which has both antibacterial and antifungal activity, further contributes to the protective environment of the placenta (26). Other natural peptides with broad activity against both Gram-positive and Gram-negative bacteria as well as yeasts, identified in the placenta, include a fragment of the human β-chain of hemoglobin (hHEMb111–146) and a fragment of human GAPDH (hGAPDH2-32). Their expression has been observed in healthy, uncomplicated pregnancies and is therefore thought to be part of the basal innate defense strategies, although information remains limited (117, 118). Principal AMPs produced in the placenta are summarized in **Table 1**.

**Table 1:** Key antimicrobial peptides in the placenta and their microbicide spectrum.

Antimicrobial peptide	Localization	Probed activity	References
HBD-1	Expressed in placental and chorion trophoblast, amnion epithelium, decidua, and amniotic fluid.	Broad-spectrum antimicrobial against bacteria and viruses; protects maternal-fetal interface from infections.	(18, 106, 138, 146)
HBD-2	Localized in amnion epithelium, chorion trophoblast, decidua, and placental trophoblast.	Antimicrobial activity against bacteria like GBS and E. coli; suppresses bacterial invasion and enhances innate immune defense.	(138, 167)
HBD-3	Expressed in placental and chorion trophoblast, amnion epithelium, and decidua.	Broad antimicrobial spectrum; prevents bacterial ascent and contributes to fetal protection during pregnancy.	(61, 106, 138)
HBD-4	Limited evidence in placenta; primarily expressed in endometrium during proliferative phase.	Antimicrobial activity in reproductive tract; role in placental defense not well-established.	(76, 79, 130)
LL-37	Expressed in human trophoblasts and placental tissue.	Broad-spectrum activity against bacteria, viruses, and fungi; vitamin D-induced antimicrobial response.	(106, 168, 169)
Lysozyme	Expressed by human placenta and fetal membranes.	Antimicrobial enzyme contributing to innate defense against bacterial infections during pregnancy.	(106, 107)
Lactoferrin	Expressed by cytotrophoblasts; their placental expression is increased in the presence of activated macrophages. Also present in amniotic fluid, and female reproductive tract.	Iron-binding antimicrobial; prevents infections and inflammation in pregnancy.	(108–111)
Hepcidin	Present in human trophoblast and amniotic fluid	Antibacterial. Also, potential role in pregnancy complications like preeclampsia	(112, 113)
SLPI	Expressed in endometrium, conceptus tissues, and trophoblast.	Antibacterial and antiviral activity; anti-inflammatory effects during pregnancy.	(107, 169, 170)
Elafin	Expressed by trophoblasts, skin, respiratory tract, intestine and endometrium, and immune cells like neutrophils and macrophages	Antibacterial and antifungal activity	(106)
LEAP-2	Limited placental-specific evidence; detected in serum during pregnancy.	Antimicrobial peptide; potential role in pregnancy complications like preeclampsia, but placental activity not well-probed.	(171, 172)

GBS, Group B Streptococcus. LEAP-2, Liver-Expressed Antimicrobial Peptide 2. SLPI, Secretory Leukocyte Protease Inhibitor.

#### 3.3.1 Placental AMPs in intrauterine infection

Maternal infections significantly alter the expression of AMPs in the human placenta; their modulation by pathogen-specific signals underscores their critical role in maintaining placental integrity and preventing vertical transmission. In response to infection, the placenta upregulates defensins, S100A9, lactoferrin, and SLPI, strengthening the barrier function and limiting pathogen invasion through cytokine-driven innate immune responses, as demonstrated with human *ex vivo* placental villi and clinical samples (119–121).

Several AMPs counteract viral pathogens that threaten placental function. Cytomegalovirus alters defensin expression and is directly inhibited by lactoferrin, which interferes with its replication (122). Multiple experimental approaches demonstrated that LL-37 binds viral envelope proteins, preventing fusion and entry (26), while HBD-2 blocks infection by binding viral glycoproteins (18, 26). SLPI has antiviral activity against herpes simplex virus (HSV) (32). Additionally, interferon-stimulated genes, such as ISG15, are upregulated during viral infection, tagging viral proteins for degradation (123). Zika virus infection is also associated with altered AMPs expression, reinforcing the antiviral shield at the maternal–fetal interface (32).

As noted, LPS is a pathogen-associated molecular pattern that strongly upregulates AMPs expression. This is true for most, but not all, AMPs. Specifically, *E. coli* LPS represses LL-37 while inducing S100A9 and HBD2 expression in cultured trophoblasts. Interestingly, calcidiol treatment counteracts this repression by stimulating LL-37 and further enhancing S100A9 expression. Conversely, cyclic AMP (cAMP), produced in response to some microbial stimuli, antagonizes vitamin D–dependent AMPs upregulation by suppressing calcitriol biosynthesis (119). Therefore, increased cAMP production and exposure to LPS may represent bacterial strategies to evade placental defenses, particularly under conditions of vitamin D deficiency during pregnancy.

Moreover, cAMP exhibits sexually dimorphic effects. In human placentas from female fetuses, cAMP correlates positively with S100A9 and defensins, providing enhanced protection against UTIs (124). In contrast, male placentas, show lower TNF-α, higher IL-10, and reduced AMP expression, indicating a sexually dimorphic weaker basal immune state (125).

Beyond infections, conditions such as gestational diabetes or preeclampsia may impair placental AMPs production and effectiveness. *In vitro* studies show that high glucose levels promote villous inflammation, reduce HBD-1, -2, -3, and -4 synthesis, and consequently increase susceptibility to infection by *E. coli* or *S. agalactiae* (66, 126). Women with late-onset preeclampsia have higher serum levels and placental gene expression of HBD-1 compared with controls, suggesting a potential protective role in mitigating the exacerbated proinflammatory response characteristic of this condition (56).

Altogether, the available evidence positions the placenta as a highly active immunological tissue that integrates hormonal, metabolic, and microbial cues to regulate AMPs production. By combining physical barrier properties with a diverse antimicrobial repertoire, the placenta plays a decisive role in limiting pathogen transmission and maintaining a protective environment for the developing fetus.

This coordinated regulation is not limited to the placenta; other extraembryonic tissues also play essential antimicrobial roles.

### 3.4 Fetal membranes

Beyond serving as physical barriers, the fetal membranes (amnion and chorion) are metabolically and immunologically active tissues that help maintain the integrity of the amniotic environment while sensing and responding to microbial challenges. In addition to producing growth factors and cytokines, these tissues synthesize and release AMPs as part of a tightly regulated defense mechanism. The amniotic epithelial, trophoblastic, and mesenchymal cells in the fetal membranes, as shown in studies using human fetal membrane tissues and *ex vivo* approaches, contribute to both constitutive and inducible AMPs production in response to inflammatory or infectious stimuli (79, 127).

This innate immune function is anatomically coordinated. The amniotic epithelium and chorionic trophoblast–stroma create spatial and biochemical gradients that enable compartmentalized antimicrobial responses directed toward the amniotic cavity or the choriodecidual interface. In ex vivo two-compartment culture models of human amniochorion challenged with live *Escherichia coli*, Olmos-Ortiz et al, demonstrated that this spatially organized defense induces side-specific AMP expression across the membrane layers (52).

Among the AMPs expressed in fetal membranes, HBD-1, HBD-2, and HBD-3 are the most extensively characterized. The amnion expresses these peptides under both basal and stimulated conditions, with HBD-3 particularly abundant and inducible by microbial or inflammatory environments, underscoring its role as a sentinel molecule at the fetal-maternal interface, as consistently reported in human-derived tissues (128). In an *ex vivo* model preserving amnion-choriodecidua polarity, García-López et al. demonstrated that *E. coli* triggers side-specific secretion of HBD-1, HBD-2, and HBD-3, predominantly from the maternal choriodecidual side, confirming the spatial coordination of AMP-mediated antimicrobial defense (129). Similarly, Zaga-Clavellina et al. observed that *Candida albicans* stimulation induces distinct secretion patterns of HBD-1, HBD-2, and HBD-3 between the amnion and choriodecidua, reinforcing the concept of asymmetric, pathogen-dependent AMPs responses in studies performed with human fetal membrane explants (130). Clinically, HBD-3 is a physiological constituent of the amniotic fluid and increases during labor and intra-amniotic infection, supporting its protective role in intra-amniotic host defense (131).

Beyond β-defensins, the cathelicidin hCAP18/LL-37 is expressed in fetal membranes and the myometrium, where inflammatory mediators upregulate its expression. Experimental exposure of human fetal membrane explants to LL-37 increases secretion of proinflammatory cytokines and matrix metalloproteinases, linking antimicrobial activation to extracellular matrix remodeling processes involved in membrane weakening at term (132).

Protease-inhibitor AMPs further strengthen this innate barrier. SLPI is synthesized by amniotic and chorionic tissues and is consistently detected in amniotic fluid, forming a baseline antimicrobial shield that persists even in the absence of infection. Its expression increases with cytokine stimulation, providing additional protection during inflammation (133, 134). Similarly, elafin is expressed in fetal membranes and upregulated under pathological conditions such as preterm premature rupture of membranes (PPROM), where it contributes to the antimicrobial and anti-protease defense of the chorioamniotic interface in studies based on human gestational tissues (135).

In addition to defensins, cathelicidin, and protease inhibitors, the fetal membranes and amniotic fluid contain a broader AMPs repertoire, including RNase-7, RNase-8, lactoferrin, psoriasin (S100A7), and lysozyme, which provide continuous antimicrobial surveillance. These peptides are expressed basally, maintaining a constitutive defense at the amnio-fluid boundary, but their secretion can be further increased by lipopolysaccharides or inflammatory cytokines such as IL-1β, as observed in human *ex vivo* membranes and clinical samples (52, 136).

In summary, the fetal membranes are structured, dynamic, and adaptable antimicrobial barriers whose compartmentalized and inducible secretion of peptides such as β-defensins, LL-37, SLPI, elafin, RNases, lactoferrin, and lysozyme is essential for protecting the intrauterine environment and maintaining membrane integrity. Dysregulation or premature activation of these AMPs may compromise this barrier, contributing to conditions such as chorioamnionitis, PPROM, and preterm labor. From a translational perspective, quantifying AMPs such as HBD-2, HBD-3, SLPI, and lactoferrin in membrane tissue or amniotic fluid is emerging as a promising diagnostic strategy for detecting intra-amniotic infection and assessing preterm birth risk, although standardization and multimarker validation are still needed (127, 131).

### 3.5 Amniotic fluid

Amniotic fluid surrounds the fetus during pregnancy, serving as a reservoir of nutrients and growth factors essential for fetal development. It also protects the fetus from physical harm and contributes to immune defense through leukocytes and soluble components (137). Among these, AMPs are particularly important, acting as a first line of defense against pathogens and as immunomodulators. AMPs provide rapid, broad-spectrum antimicrobial activity, functioning even before the fetal immune system is fully developed. Unlike other immune components, AMPs are continuously present in amniotic fluid, ensuring sustained protection against pathogens and excessive immune responses from the fetus (106, 138).

AMPs in amniotic fluid originate from fetal, placental, and maternal tissues. Their presence and concentration can be influenced by factors such as gestational age, maternal health (especially infections), and fetal stress (79, 106, 139, 140). Secretion of AMPs into amniotic fluid is highly regulated and varies across different stages of pregnancy. In early pregnancy, the amniotic cavity forms by the fourth week of gestation. During this initial phase, amniotic fluid consists primarily of water and electrolytes. The concentration of AMPs is relatively low at this stage and is scarcely documented due to limited opportunities to safely access the amniotic cavity (141).

As pregnancy progresses into the second and third trimesters, the risk of ascending infections increases, mainly due to physiological changes in the cervix as it prepares for labor. These changes can facilitate the ascent of pathogens from the lower genital tract into the amniotic cavity, posing significant risks to both mother and fetus (142). However, multiple studies *in vitro* have demonstrated the strong antibacterial activity of amniotic fluid against a wide range of microorganisms associated with urinary and cervico-vaginal infections (e.g., *E. coli, S. agalactiae, C. albicans*) as well as wound-associated pathogens (*E. faecium, S. aureus, S. pyogenes K. pneumoniae, A. baumannii, P. aeruginosa, and E. aerogenes*), without affecting beneficial and probiotic microbiota strains such as *Lactobacillus plantarum* (134, 143–145).

During this period, β-defensins are the main AMPs studied for their role in preventing and resolving infections. Amniotic fluid HBD-1 levels increase significantly in mid-pregnancy, reaching an average concentration of 13.7 ng/mL. However, their concentration decreases slightly in the third trimester, averaging 9.1 ng/mL (146). This suggests that HBD-1 may serve as a more prominent role as a soluble defense mechanism in mid-pregnancy than in later stages within the amniotic cavity. In contrast, the median concentration of HBD-2 remains relatively stable, with values of 3 ng/mL during the second trimester and 2.9 ng/mL at term. This stability indicates that HBD-2 levels do not change significantly throughout a healthy pregnancy. Although HBD-2 is typically considered as an inducible molecule in response to inflammation and infection, its consistent presence in amniotic fluid during normal pregnancy suggests a potential role in maintaining basal fetal immunity (147).

The available literature provides limited data on the specific concentrations of alpha defensins. Because HNP 1–3 are primarily expressed by neutrophils, their presence in amniotic fluid is likely predominantly of fetal origin. Amniotic fluid concentrations of these peptides increase in both preterm and term gestations, suggesting that, in addition to their protective role, they are also involved in labor (148).

Information on normal levels of LL-37 in amniotic fluid is not yet well-established. However, some studies have confirmed its presence in the amniotic cavity using Western blot analysis (149). Further research is needed to fully understand how LL-37 concentrations in amniotic fluid fluctuate throughout normal and complicated pregnancies.

In addition to its ability to protect fetal tissues by inhibiting proteolytic enzymes, SLPI also has anti-inflammatory properties that help maintain a balanced immune response during gestation (86, 150). Notably, SLPI levels in amniotic fluid increase significantly from 106 ng/mL in the second trimester to 802 ng/mL in the third trimester, indicating a substantial rise in SLPI production as pregnancy progresses, and suggesting it may be the most significant protector of the fetus during gestation (133).

Although specific data on Elafin/Trappin-2 concentrations in amniotic fluid throughout human pregnancy are lacking, its presence in other reproductive tissues and its role in immune response during pregnancy are well-documented (151). Further research is needed to determine elafin’s presence and concentration changes in amniotic fluid during pregnancy, but existing evidence highlights its potential importance in fetal defense mechanisms and immunoregulation.

These findings indicate that the continuous production of these AMPs throughout pregnancy, along with their non-harmful mechanism of action, protects against pathogens without triggering significant inflammatory responses. Their stability during pregnancy is essential for maintaining a constant, low-level barrier against infection, ensuring continuous protection of the intrauterine environment without overstimulating the immune system.

#### 3.5.1 Changes in amniotic fluid AMPs during infection

Infections during pregnancy, especially those affecting the amniotic cavity, pose significant risks to both mother and fetus. AMPs play a crucial role in host defense against microbial invaders. Several studies have shown that AMPs levels in amniotic fluid change in the presence of infection, which can lead to PPROM, microbial invasion of the amniotic cavity (MIAC) or histologic chorioamnionitis (HCA), reflecting activation of the innate immune response. HCA is defined as histopathological evidence of infection or inflammation in the amnion or chorion.

β- and α- defensins: HBD-2 concentrations increase in the amniotic fluid of women with MIAC, regardless of whether the membranes are intact or ruptured. Similarly, HBD-1 has been reported at elevated levels in women with spontaneous preterm birth and intra-amniotic infection (146, 147, 152). HNP 1–3 also rise markedly in the presence of MIAC and HCA (148). Their presence and infection-driven upregulation suggest a protective role for the fetus and highlight their potential diagnostic utility. These peptides can also increase during PPROM. Importantly, α-defensins, which are largely (>98%) derived from endometrial neutrophils, have been investigated as surrogate biomarkers of clinical and subclinical chorioamnionitis and fetal inflammation, with or without infection. Conversely, low defensin levels in cord blood have been associated with an increased risk of late-onset sepsis (153).

Cathelicidin: A study of women with PPROM found that LL-37 concentrations in amniotic fluid were significantly higher in those with MIAC and HCA compared to those without these complications. Specifically, the study reported a median amniotic fluid concentration of LL-37 of 3.6 ng/mL in women with MIAC and HCA, while women without these conditions had a median concentration of 1.4 ng/mL. Additionally, a cutoff of 4.0 ng/mL showed high specificity (90%) for identifying women with MIAC and HCA, indicating its value for detecting patients at high risk of inflammatory complications (154). These findings suggest that cathelicidin could serve as a potential biomarker for identifying patients with infection-related inflammatory complications.

Lactoferrin levels have also been reported to be elevated in the amniotic fluid of patients with HCA, showing a positive and significant correlation with amniotic fluid IL-6 levels (155, 156).

AMPs levels in amniotic fluid have significant clinical implications, particularly for intra-amniotic infections and preterm labor, with potential as diagnostic and therapeutic tools. Available evidence suggests that certain AMPs may increase in amniotic fluid in response to microbial invasion, highlighting their potential as biomarkers of intra-amniotic infection. Early diagnosis of intra-amniotic infection allows timely medical intervention, which may include antibiotics or measures to delay preterm labor, leading to improved neonatal outcomes and reducing the risk of complications such as neonatal sepsis, bronchopulmonary dysplasia, and brain injury. Importantly, the antimicrobial protection within the amniotic environment is also complemented by AMP production in fetal tissues.

### 3.6 Fetal tissues

During fetal and early postnatal life, defensins are the most significant AMPs that protect and support proper fetal development. To reduce the risk of infection, several fetal organs, including the skin, lungs, and gut, actively express various AMPs.

Fetal skin is thin and vulnerable to pathogens, increasing the risk of infection. As a defense mechanism, fetal skin constitutively produces HBD-2, HBD-3, S100 protein family members, LL-37, and low levels of RNAse-5 and RNAse-7 through reduced histone methylation mechanisms (157). In response to inflammatory IL-1α or bacterial stimuli, the fetal skin and vernix caseosa can also synthesize defensins, LL-37, HNP-1-3, SLPI, lactoferrin, lysozyme, and calprotectin, which display antimicrobial activity against both Gram-positive and Gram-negative bacteria, such as Group B *Streptococcus*, *Escherichia coli, Bacillus megaterium*, and the yeast *Candida albicans* (106, 158, 159). Other less studied antimicrobials in vernix caseosa include ubiquitin, palate lung nasal epithelial clone (PLUNC), neutrophil gelatinase-associated lipocalin (NGAL), cystatin A, and uteroglobin-related protein 1 (UGRP-1) (159, 160). Moreover, S100A7 and RNase-7 concentrations increase significantly in neonatal skin over time; therefore, preterm newborns have lower baseline levels of these peptides, which are thought to contribute to the first line of defense against infection. Nevertheless, despite their lower levels compared with full-term infants, preterm neonates are still capable of inducing antimicrobial peptide synthesis in response to infection, as observed in cases of HCA (55).

On the other hand, HBD-2 is the predominant defensin in the fetal pulmonary epithelium (161) and remains so during the neonatal period (62). HBD-1 is also expressed in the neonatal lung, but to a lesser extent, while LL-37 and HBD-3 are not detected during this period (62). The lungs and gastrointestinal tract of the fetus produce defensins and LL-37 in response to infectious stimuli (161, 162).

In the fetal gut, during the second trimester of gestation, there is a progressive increase in the density of immune-competent Paneth-cells, which produce large amounts of HD-5 and HD-6, particularly in the ileum (163, 164). Premature infants have a limited number of Paneth cells, which also show reduced capability to produce AMPs (165). This may explain the higher rates of necrotizing enterocolitis (NEC) in preterm infants. This disease is caused by bacterial invasion and inflammation of the intestinal wall. Neonates with NEC show upregulation of HD-5 and HD-6 mRNA levels in Paneth cells (162); however, HD-5 and HD-6 overexpression cannot compensate for gut bacterial translocation. Low fecal and intestinal hBD-2 levels have been associated with severe NEC, highlighting a possible preventive effect (166).

By modulating inflammation and shaping early immune responses, AMPs influence immune maturation, neurodevelopment, nutrient absorption, and microbiome establishment. For example, β-defensins are induced in fetal brain cells by bacterial components, highlighting their importance in protecting against neuronal bacterial pathologies (63).

## 4 Concluding remarks

From the beginning to the end of pregnancy, the fetus coexists within the uterus, where, despite employing multiple immunological and endocrine strategies to evade rejection, it remains a semi-allogeneic entity within the maternal environment. Therefore, the intimate interaction between maternal and fetal tissues requires tightly regulated mechanisms capable of detecting and counteracting signals that could disrupt the delicate equilibrium of immune privilege. Critically, this protection does not rely on a single structure but rather on the coordinated function of all tissues at the maternal–fetal interface, which together form an integrated barrier designed to shield the fetus from potential pathogens.

A key strategy supporting this collective defense is the action of the innate immune system as a central controller of protective responses. Within this system, AMPs serve as versatile and tissue-specific effectors that can be rapidly secreted to counter microbial threats that may compromise the continuity of gestation.

The evidence presented here highlights AMPs as an intrinsic and effective layer of defense; however, they remain only one component of a broader and inherently limited protective network. Therefore, advancing our understanding of their mechanisms and exploring their potential as therapeutic targets may provide an opportunity to reinforce the MFI and ultimately delay or prevent multiple adverse pregnancy outcomes.

This review has several limitations that should be acknowledged. First, as a narrative synthesis, it does not formally assess inter-study heterogeneity which may limit the ability to weigh the relative strength and consistency of the evidence. Second, the available literature is highly heterogeneous in terms of study design, experimental models, gestational stages, and biological compartments analyzed, which complicates direct comparisons across studies. Third, a substantial proportion of the evidence derives from *in vitro* systems or *ex vivo* tissue models, which may not fully recapitulate the complexity of *in vivo* conditions during human pregnancy. Fourth, reported AMP concentrations and expression patterns vary depending on methodological approaches, limiting cross-study standardization. Finally, although this review focuses on human pregnancy, variability related to maternal factors, such as metabolic status, microbiota composition, nutritional status, and environmental exposures, remains insufficiently characterized, highlighting the need for more integrative and longitudinal studies.
